# Is Nerve Electrophysiology a Robust Primary Endpoint in Clinical Trials of Treatments for Diabetic Peripheral Neuropathy?

**DOI:** 10.3390/diagnostics12030731

**Published:** 2022-03-17

**Authors:** Dalal Y. Al-Bazz, Andrew J. Nelson, Jamie Burgess, Ioannis N. Petropoulos, Jael Nizza, Anne Marshall, Emily Brown, Daniel J. Cuthbertson, Andrew G. Marshall, Rayaz A. Malik, Uazman Alam

**Affiliations:** 1 Institute of Life Course and Medical Sciences and the Pain Research Institute, University of Liverpool and Liverpool University Hospital NHS Foundation Trust, Liverpool L9 7AL, UK; a.j.nelson@liverpool.ac.uk (A.J.N.); jamie.burgess@liverpool.ac.uk (J.B.); jael.nizza@nhs.net (J.N.); anne.marshall@liverpool.ac.uk (A.M.); andrew.marshall@liverpool.ac.uk (A.G.M.); 2 Research Division, Weill Cornell Medicine-Qatar, Qatar Foundation, Education City, Doha 24144, Qatar; inp2002@qatar-med.cornell.edu (I.N.P.); ram2045@qatar-med.cornell.edu (R.A.M.); 3 Obesity and Endocrinology Research Group, Institute of Life Course and Medical Sciences, University of Liverpool and Liverpool University Hospital NHS Foundation Trust, Liverpool L9 7AL, UK; c.e.brown@liverpool.ac.uk (E.B.); dan.cuthbertson@liverpool.ac.uk (D.J.C.); 4 Institute of Cardiovascular Sciences, Cardiac Centre, Faculty of Medical and Human Sciences, University of Manchester and NIHR/Wellcome Trust Clinical Research Facility, Manchester M13 9WL, UK; 5 Division of Diabetes, Endocrinology and Gastroenterology, Institute of Human Development, University of Manchester, Manchester M13 9PL, UK

**Keywords:** nerve electrophysiology, peripheral neuropathy, diabetes

## Abstract

There is currently no FDA-approved disease-modifying therapy for diabetic peripheral neuropathy (DPN). Nerve conduction velocity (NCV) is an established primary endpoint of disease-modifying therapies in DPN and clinical trials have been powered with an assumed decline of 0.5 m/s/year. This paper sought to establish the time-dependent change in NCV associated with a placebo, compared to that observed in the active intervention group. A literature search identified twenty-one double-blind, randomised controlled trials in DPN of ≥1 year duration conducted between 1971 and 2021. We evaluated changes in neurophysiology, with a focus on peroneal motor and sural sensory NCV and amplitude in the placebo and treatment groups. There was significant variability in the change and direction of change (reduction/increase) in NCV in the placebo arm, as well as variability influenced by the anatomical site of neurophysiological measurement within a given clinical trial. A critical re-evaluation of efficacy trials should consider placebo-adjusted effects and present the placebo-subtracted change in NCV rather than assume a universal annual decline of 0.5 m/s/year. Importantly, endpoints such as corneal confocal microscopy (CCM) have demonstrated early nerve repair, whilst symptoms and NCV have not changed, and should thus be considered as a viable alternative.

## 1. Introduction

The prevalence of diabetic peripheral neuropathy (DPN) is estimated to be ~50% but may be higher depending on the duration and type of diabetes [1]. Around 30% of people with diabetes experience symptoms which may be positive (tingling, burning) and/or negative (numbness) [2,3], affecting the lower limb [4]. Although the optimization of glycaemic control and cardiovascular risk factors is recommended to limit the development or progression of diabetic neuropathy [5,6,7], regrettably, there are no currently approved (US Food and Drug Administration (FDA), or European Medicines Agency (EMA)) disease-modifying treatments for DPN [8].

The efficacy of several pathogenetic treatments, including aldose reductase inhibitors, antioxidants, and protein kinase C inhibitors, has been assessed in clinical trials over the past four decades [9]. All have failed in phase III clinical trials with none approved as treatments for DPN by the either the FDA or EMA [10,11]. Numerous reasons have been cited for these failures, including a lack of drug efficacy, trial duration and inappropriate endpoints. Composite clinical scores and quantitative sensory testing rely on patient responses and are deemed to be subjective [12] and prone to high variability. The results of neurophysiology-based assessments continue to be endorsed as a primary endpoint in clinical trials of DPN [13,14,15], despite a high inter-observer variability [16]. Most clinical trials are powered assuming a nerve conduction velocity (NCV) decline of 0.5 m/s/year in the placebo group [17,18]. However, the overall improvement in glycaemic control (with safer, more effective glucose lowering therapies) and a wider use of ACE inhibitors and statins over the last two decade will have modified the natural history of DPN. Thus, the application of this (exaggerated) rate of decline could lead to false interpretations in treatment effects and rejection of the approval of effective treatments for DPN. To this point, Dyck et al. [19] showed an enhanced placebo effect on symptoms and signs and much slower NCV reduction when assessing the natural history of DPN in the placebo arms of two large clinical trials and the Rochester DPN epidemiological study [19,20].

To objectively address the issue of the variable rate of decline in NCV, we have evaluated the change in NCV in the placebo and treatment arms of trials of pathogenetic treatments for DPN with a duration ≥12 months.

## 2. Methods

Electronic literature searches of the MEDLINE database were performed for trials of pathogenetic treatments in diabetic neuropathy. The searches were restricted to the English language from January 1971 to July 2021.

Authors D.Y.A. and A.J.N. independently searched the stated databases using combinations of the following search terms: “α-lipoic acid”, “AcetylCarnitine”, “Acetyl-L-Carnitine”, “Alrestatin”, “ARI AS-3201”, “Clofibrate”, “C-peptide”, “Diabetes”, “Diabetes mellitus”, “Diabetes mellitus, type 1”, “Diabetes mellitus, type 2”, “Diabetic”, “Epalrestat”, “Findarestat”, “Gamma-Linolenic acid”, “Gangliosides”, “Glibenclamide”, “Insulin”, “Intensive glycaemic therapy”, “Intensive glycemic therapy”, “Levacecarnine”, “Linolenic acid”, “Myoinositol”, “myo-Inositol”, “Neurotrophic peptide”, “ORG 2766”, “ORG-2766”, “Peripheral Neuropathy”, “Plasmid”, “PKCI”, “Ponalrestat”, “Pyridoxine”, “protein kinase c”, “Ranirestat”, “rhNGF”, “Ruboxistaurin”, “Sorbinil”, “Sulodexide”, “T1DM”, “T2DM”, “Thioctic acid”, “Tolrestat”, “Topiramate”, “Trandolapril”, “vascular endothelial growth factor”, “VEGF”, “Vitamin E”, “Zenarestat”, “Zinc ADJ6” and “Zinc Sulphate”.

All search results were combined using Endnote X7.8 and duplicates removed. Reference lists of the primary and secondary literature were browsed to identify additional studies and hand searching of reference lists were also performed.

Peroneal motor NCV and sural sensory NCV and amplitude were extracted to an electronic spreadsheet and the change analysed in the placebo and treatment arms.

### Inclusion and Exclusion Criteria

Studies were included in the final evaluation based on an a priori inclusion criteria. For inclusion, studies were required: (1) to be a double-blind, randomised controlled trials, (2) to be a minimum of one-year in duration, and (3) to have neurophysiology included as an endpoint (4) in diabetic peripheral neuropathy.

Studies were excluded if they: (1) were not a human study, or (2) not in the English language.

**Figure 1:**
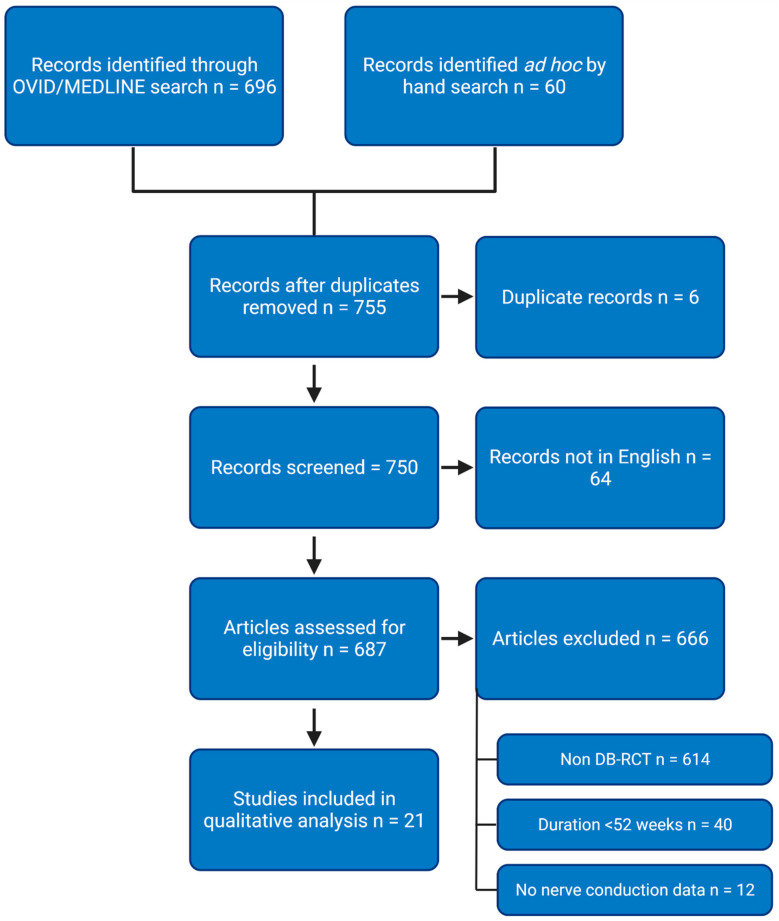
Search strategy. DB-RCT, double-blind randomised control trial. Figure created with https://BioRender.com (accessed on 3 February 2022).

A.J.N. screened all articles and selected those that satisfied the inclusion criteria for full article evaluation. This was then subsequently reviewed by D.Y.A. All titles and abstracts of articles were screened to remove irrelevant studies, and the remaining shortlisted articles were screened for eligibility in depth. The full texts of relevant articles were retrieved, screened, and selected using the inclusion and exclusion criteria (Figure 1). The original authors were not contacted for additional data. Any concerns regarding inclusion or exclusion were decided by the senior author (U.A). Before data extraction, U.A. assessed all articles to confirm their eligibility in the study. Our study partly utilises a systematic review methodology, although it was primarily designed as a standard review of the literature. A meta-analysis/summary data synthesis was not undertaken due to an a priori decision that the quality of data reporting from included studies was likely to be poor. The study or study protocol was therefore not registered with a systematic review database such as PROSPERO.

## 3. Results

Twenty-one studies published between 1988 and 2016 met the a priori inclusion criteria (Table 1), with an average duration of 67.9 ± 37.3 weeks (median 52 weeks; IQR 52–64.5 weeks) (placebo: *n* = 2395, treatment: *n* = 3541). The available study characteristics and demographic data are presented for each study in Table 2. The populations recruited had a heterogeneity of diabetes sub-type (type 1 and type 2 diabetes), with more participants with type 2 diabetes being enrolled in later years. A range of putative treatments were studied: aldose reductase inhibitors (ARI), protein kinase C beta (PKC-β) inhibitors, amino acid/Carnitine acetyltransferase, nerve growth factor—rhNGF, neurotrophic peptide/ACTH 4-9 analogue, antioxidants, angiotensin-converting-enzyme (ACE) inhibitors, ɣ-Linolenic acid (GLA) and C-peptide.

Trials evaluated between 1980 and 2009 with available NCS data (*n* = 19), both peroneal motor NCV and sural sensory NCV exhibited an increase in the placebo arm (+0.17 ± 0.77 m/s/year and +0.09 ± 1.31 m/s/year, respectively), while sural amplitude showed a marginal reduction (−0.11 ± 0.48 uV/year). These results are consistent with those from the most recent trial undertaken [21] which demonstrated an increase in peroneal motor NCV and sural sensory NCV (+0.3 ± 0.2 m/s/year and +1.2 ± 0.29 m/s/year) with a slight reduction in sural amplitude (−0.2 ± 0.2 mV/year) in the placebo group [21]. A further trial demonstrated a marginal reduction in peroneal motor NCV at 4 years (−0.06 ± 4.07 m/s) [22].

### 3.1. Protein Kinase C Beta (PKC-β) Inhibitor—Ruboxistaurin (RBX)

PKCβ is activated by hyperglycaemia and disordered fatty acid metabolism, and it is involved in the pathogenesis of endothelial damage in diabetes. Ruboxistaurin (RBX), a specific inhibitor of PKCβ, showed favourable results in animal models and in short-duration clinical trials of diabetic neuropathy [23,24].

Brooks et al. demonstrated the impact of RBX 32 mg/d versus placebo on skin microvascular blood flow (SkBF) and sensory symptoms in a small cohort of patients with DPN (*n* = 11 placebo, *n* = 9 Ruboxistaurin) [25]. Peroneal motor NCV improved significantly in the placebo (median: +0.5 m/s (IQR: 0.2 to 1.7) compared to a decrease with RBX (median: −1.2 (IQR: −2.4 to 0.1) (*p* = 0.034) [25]. Tibial motor NCV declined less (median: −0.5 m/s (IQR: −1.3 to 2.4) and amplitudes increased (median: 0.9 mV (IQR: −0.6 to 1.4) in the placebo compared to RBX (median NCV: −1.4 m/s (IQR: −2.5 to 2.9; median amplitude: −1.3 mV (IQR: −1.8 to 0.7) group, although the difference was not significant. Sural sensory NCV declined in both the placebo (median: −0.9 m/s (IQR: −2.7 to 1.8) and RBX (median: −0.6 m/s (IQR: −2.7 to 1.8) groups which was not significant. There was no effect of RBX on endothelium-dependent, endothelium-independent, or C-fibre-mediated change in SkBF or sensory symptoms (NTSS-6 total score: 7.7 vs. 6.0 points; *p* = 0.4) and there was no correlation between change in nerve conduction and SkBF [25].

Tesfaye et al. evaluated the effect of RBX and placebo on disease progression and neuropathic symptoms in two one-year clinical trials in patients with mild DPN [26]. The placebo groups (*n* = 262, with *n* = 211) demonstrated an improvement in symptoms with a decline in peroneal motor NCV (−0.38 ± 2.2 m/s, *p* = 0.012), and sural nerve amplitude (−1.12 ± 3.7, *p* < 0.001), although, paradoxically, the tibial F-wave latency also declined (−0.33 ± 2.4, *p* = 0.045) [26]. The authors concluded that worsening of DPN in placebo-administered patients requires >1 year of observation [26].

Vinik et al. showed that neither 32 mg/d nor 64 mg/d of RBX altered the vibration detection threshold (VDT) or Neuropathy Total Symptom Score (NTSS-6) compared to placebo and electrophysiological data were not presented [27]. In patients with significant symptomatic DPN at baseline (NTSS-6 total score >6; *n* = 83), there was a significant improvement in the NTSS-6 total score over 12 months in the RBX 64 mg/d treatment arm compared to placebo (*p* = 0.015). A subgroup of participants with clinically significant symptoms (NTSS-6 total score >6) and less severe DPN (sural sensory nerve action potential >0.5 uV) demonstrated a significant change in the NTSS-6 total score with RBX 64 mg compared to placebo (−5.25 vs. −1.61 points; *p* = 0.006) [27].

### 3.2. Aldose Reductase Inhibitors (ARI)

Aldose reductase coverts glucose to sorbitol in the polyol pathway and sorbitol accumulation in the peripheral nerve is associated with DPN. A total of six aldose reductase inhibitors have been developed [28] and are the most widely investigated disease-modifying therapies for DPN [29,30,31,32,33,34,35,36,37,38,39].

#### 3.2.1. Sorbinil

The Sorbinil retinopathy trial was conducted in patients with type 1 diabetes (*n* = 497) in the mid 1980s and found that approximately 30% of patients had worsening clinical measures of symmetric distal neuropathy in both the Sorbinil-treated and placebo arms [29]. Only 192 of 497 patients had NCS and there was no significant difference in the change in NCV or amplitude in the two groups [29]. There was also a trend for NCV to decline in the Sorbinil group, although the peroneal motor NCV improved in the Sorbinil group (+0.5 ± 4.4 m/s) and declined in the placebo group (−0.9 ± 3.5) (*p* = 0.02). The median motor NCV (−0.8 ± 4.1), sensory forearm NCV (−1.7 ± 7.5), sensory distal NCV (1.1 ± 7.0) and peroneal motor NCV (−0.9 ± 3.5) declined in the placebo arm [29]. A regression model demonstrated that higher baseline nerve conduction velocity and amplitudes were associated with a greater decrease over time [29].

In a two-year study of patients with severe symptomatic DPN (*n* = 21) [30], there was no significant change between the Sorbinil or placebo groups for 12 neurophysiological parameters [30]. However, the study had an attrition rate of 33%, due to adverse reactions and only 14 patients completed the trial (*n* = 8; Sorbinil, *n* = 6; placebo). Similarly, in a double-blind trial of Sorbinil 250 mg daily over 12 months in patients with DPN, there was no change in symptoms or neurophysiology compared to placebo and, paradoxically, neuropathic diabetic foot ulcers developed in 19% of patients in the Sorbinil group (*n* = 21) compared to 10% in the placebo group (*n* = 10) [31].

#### 3.2.2. Ranirestat

Bril et al. investigated the effect of multiple doses of Ranirestat (10, 20 or 40 mg/day) and placebo over 1 year in patients with mild-to-moderate DPN [32]. There was no difference in sensory NCS vs. placebo, but there was an improvement in the summed motor NCV of the peroneal, tibial, and median nerves in the higher doses at weeks 12, 24, and 36 (*p* ≤ 0.05), and the peroneal NCV at 36 and 52 weeks (*p* ≤ 0.05) for intermediate dosing. At week 52, the summed sensory NCV (bilateral sural and proximal median sensory) increased in both the placebo (+2.0 m/s) and Ranirestat (+3.2–3.8 m/s) group, with no significant difference between groups.

#### 3.2.3. Zenerastat

In a 52-week clinical trial, Zenerastat was associated with an increase in sural NCV, composite rank score (*p* = 0.004) and density of sural nerve myelinated fibres <5 microns [33]. Peroneal, median, and sural NCV all reduced by >0.25 m/s in the placebo group and increased by +1–1.5 m/s in the 600 mg twice-daily treatment group, with intermediate trends in the 150 and 300 mg twice-daily groups. Median NCV declined in all groups, attributed to carpal tunnel syndrome, although the decline tended to be greater in the placebo group (−1.41 ± 0.90 m/s) than in the 150, 300, and 600 mg twice-daily groups (−0.37 ± 0.61, −0.30 ± 0.67, and −0.28 ± 0.60 m/s, respectively, *p* = 0.085). Peroneal NCV tended to decrease, and the total myelinated fibre density in the largest fascicle also tended to decrease in the placebo and 150 mg twice-daily treatment groups with an increase in the 300 and 600 mg twice-daily groups (*p* = 0.105; *p* = 0.077 respectively).

A large phase-three trial in patients with mild DPN (*n* = 471), Zenerastat (600 mg/day and 1200 mg/day) showed an improvement or lack of progression in all nerve conduction study measures over 12 months. However, a significant increase in serum creatinine in several patients led to the early termination of the study and discontinuation of clinical development of Zenerastat. In the placebo group, there was a significant decline in median sensory amplitude (−0.80 ± 4.86 µV; *p* = 0.0021 [95%CI: −1.3 to −0.29)) and sural sensory NCV (−0.65 ± 3.7 m/s; *p* = 0.0008 [95%CI: −1.04 to −0.27) with a paradoxical improvement in median distal motor latency (+0.18 ± 0.92 m/s; *p* = 0.002 [95%CI: 0.09 to 0.28]) [34]. There was a non-significant improvement in peroneal motor F-wave latency (+0.30 ± 3.08 m/s) and decline in median forearm sensory NCV (−0.05 ± 3.4 m/s), median motor F-wave latency (+0.18 ± 0.92 m/s) peroneal motor NCV (−0.2 ± 2.2 m/s) and sural sensory amplitude (−0.30 ± 3.11 μV).

#### 3.2.4. Ponalrestat

Ponalrestat was studied in three high-quality RCTs in the 1990s [35,36,37]. Ponalrestat showed no improvement at 18 months (sural sensory NCV baseline: 39.53 ± 6.01 m/s, 18 months: 40.51 ± 6.13 m/s; peroneal motor NCV baseline 37.10 ± 3.46 m/s, 18 months: 37.41 ± 4.38 m/s), and the placebo arm showed a slight improvement (sural sensory NCV baseline: 38.55 ± 5.16 m/s, 18 months: 39.80 ± 4.82 m/s; peroneal motor NCV baseline: 36.89 ± 3.00 m/s, 18 months: 37.25 ± 3.49 m/s) [36]. In two further RCTs with Ponalrestat 600 mg daily, there was no significant change in symptoms and neurophysiology [35,37] or autonomic nerve function [36]. In the placebo group, Laudadio et al. found worsening in the Valsalva ratio (0.46 ± 0.19 vs. 0.39 ± 0.19; *p* < 0.01), vibration (1.51 ± 0.59 vs. 1.66 ± 0.63 U; *p* < 0.01) and thermal (0.47 ± 1.12 vs. 0.76 ± 1.22 Celsius; *p* < 0.01) perception thresholds of the lower extremities; median motor NCV (−0.35 ± 0.30 m/s), distal sensory NCV (−1.40 ± 0.43 m/s, *p* = 0.01), proximal sensory NCV (−0.57 ± 0.31 m/s), ulnar distal sensory NCV (−1.27 ± 0.55 m/s, *p* = 0.02) and sural sensory NCV (−0.89 ± 0.48 m/s). However, Peroneal motor NCV (+0.09 ± 0.26 m/s), median amplitude (+0.017 ± 0.03 mV) improved, although, paradoxically, the median F-wave latency (+0.48 ± 0.13, *p* ≤ 0.01) and peroneal F-wave latency (+1.28 ± 0.37, *p* ≤ 0.01) declined in the placebo group. Importantly, this study also suggested that based on deterioration in the placebo group, at least 250 patients needed to be treated over 2 years to demonstrate drug efficacy using electrophysiology [35].

#### 3.2.5. Fidarestat

In a 52-week study, Fidarestat 1 mg daily lead to a significant improvement in symptoms of DPN and median nerve NCV and latency [38]. In the placebo group, median F-wave conduction velocity improved (−0.6  ±  0.3 m/s; *p* < 0.001) significantly at 52 weeks with no change in median motor (−0.2  ±  0.4 m/s), median sensory (distal) (−0.1  ±  0.5 m/s), median sensory (forearm) (0.0  ±  0.5 m/s) and tibial motor (+0.1  ±  0.4 m/s) NCV [38]. The Fidarestat group demonstrated a significant improvement in tibial motor NCV (+0.8  ±  0.3 m/s, *p* < 0.0001), which was attenuated by an increase (+0.1  ±  0.4 m/s) in the placebo group. There were non-significant changes in the median motor (−0.0  ±  0.4 m/s), median sensory (distal) (+0.3  ±  0.5 m/s) and median sensory (forearm) (+0.6  ±  0.5 m/s) NCV in the treatment group [38].

#### 3.2.6. Tolrestat

Santiago et al. [39] randomly assigned patients treated with Tolrestat for an average of 4.2 years to either placebo or continued Tolrestat therapy for 52 weeks. There was a decrease (−0.9 ±  0.2 m/s) in summed NCV (median, ulnar, peroneal, and sural nerve) in the placebo group, whereas in patients who continued on Tolrestat, MNCV did not differ significantly from baseline with changes ranging from −0.6 ±  0.4 to +0.7 ±  0.5 m/s; *p* ≤ 0.05). After 3 months, patients were given the choice to switch to placebo, and they showed a significant decrease in MNCV (−1.6 ±  0.6 m/s), whilst those on placebo who switched to Tolrestat showed a significant increase in NCV (*p* < 0.05), toe sensation and pain (*p* ≤ 0.005). Tolrestat was approved in 1988 and marketed in several countries but was discontinued in 1996 due to severe liver toxicity and death.

### 3.3. Acetyl-L-Carnitine (Levacecarnine)

In a multicenter trial, 333 patients were randomized to treatment with acetyl-L-Carnitine (LAC) (1000 mg/day intramuscularly for 10 days followed by 2000 mg/day orally for 355 days) or placebo [40]. At 12 months, ulnar, sural and median sensory NCV showed a minimal decrease in peroneal and median motor NCV, whilst LAC-treated patients showed an increase in motor and sensory NCV with significant differences in peroneal amplitude (+2.2 vs. +0.1 mV), peroneal NCV (+2.7 vs. −0.2 m/s), ulnar NCV (+2.9 vs. +0.1 m/s), and sural NCV (+7 m/s vs. +1.0 m/s) (*p* ≤ 0.01) and a significant decrease in VAS score for pain in the LAC-treated group (39%) compared to placebo (8%) [40].

### 3.4. Recombinant Human Nerve Growth Factor (rhNGF)

Nerve growth factor (NGF) promotes the survival of small-fibre sensory and sympathetic neurons. A multicenter phase-three study in 1019 patients with type 1 or type 2 diabetes and DPN assessed the effects of recombinant NGF (rhNGF) 0.1 mcg/kg (*n* = 504) or placebo (*n* = 515) subcutaneously three times per week over 48 weeks [41]. The global symptom assessment (*p* = 0.03), severity of pain in the legs (*p* = 0.05) and six-month symptoms in the feet and legs (*p* = 0.003) demonstrated an improvement with rhNGF. However, sural nerve amplitude marginally decreased in the placebo (−0.1 ± 1.7 µV) and rhNGF (−0.1 ± 1.9 µV) groups, whilst the results of the median NCV and sural NCV were not reported for the study.

### 3.5. Neurotrophic Peptide/ACTH4-9/acth (4-9)-msh (4-9) Analogue

ACTH4-9 analogue (ORG 2766) 3 mg daily subcutaneous injection has been compared to placebo in a single-centre clinical trial of patients with T1DM and peripheral neuropathy (*n* = 62) [42]. There was a significant improvement in the vibration threshold in the ORG group (*p* = 0.05) with no difference in ulnar and sural sensory NCV and ulnar and tibial motor NCV. Small improvements were seen in the median NCV in both cohorts (ORG 2766: +0.50 ± 1.26 m/s, placebo: +0.89 ± 0.68 m/s). Whilst there was a decline in ulnar sensory NCV (−0.5 ± 4.3 m/s), tibial MNCV (−0.50 ± 2.98 m/s) and sural sensory NCV (−1.08 ± 5.92 m/s) in the placebo arm, there was a greater decline in ulnar sensory NCV (−2.42 ± 3.20 m/s) and sural sensory NCV (−2.12 ± 3.89 m/s) in the ORG group.

### 3.6. Antioxidants—Thioctic Acid/α-lipoic Acid

Oxidative stress plays a significant role in the pathogenesis of diabetic neuropathy [43]. α -lipoic acid is a potent antioxidant and has shown a reduction in oxidative stress and improved distal nerve conduction. Reljavonic et al. randomly assigned 65 patients with DPN and type 1 or type 2 diabetes to: (1) 2 × 600 mg of Thioctic acid (TA 1200 mg) vs. placebo, (2) 600 mg of TA vs. placebo (TA 600 mg), or (3) placebo over 24 months [44]. There was a significant change in sural sensory NCV in TA 1200 (+3.8 ± 4.2 m/s), TA 600 (+3.0 ± 3.0 m/s) and placebo (−0.1 ± 4.8 m/s) (*p* < 0.05), sural amplitude in TA 1200 (+0.6 ± 2.5 uV, (*p* = 0.08), TA 600 (+0.3 ± 1.4 uV in (*p* < 0.05) and placebo (−0.7 ±1.5 uV) and tibial motor NCV in TA 1200 (+1.2 ± 3.8 m/s), TA 600 (−0.3 ± 5.2 m/s) and placebo (−1.5 ± 2.9 m/s) (*p* < 0.05 for TA 1200 vs. placebo). There were no significant differences in tibial motor nerve distal latency and the neuropathy disability score. In the Nathan I trial, individuals with mild-to-moderate DPN were randomised to 600 mg daily of α-lipoic acid (*n* = 233) or placebo (*n* = 227) over 4 years. At 2 years, there was little change in peroneal NCV in either treatment (0.04 ± 3.89 m/s) or placebo (0.18 ± 3.99 m/s) with only a marginal reduction at 4 year follow-up (treatment: −0.35 ± 4.23 m/s; placebo −0.06 ± 4.07 m/s) [22]. Again, there were only marginal changes in sural SNAP (2 years: treatment −0.00 ± 2.17 uV, placebo −0.07 ± 1.96 uV, 4 years treatment −0.20 ± 2.34 uV, placebo −0.15 ± 2.43 uV) [22].

### 3.7. Angiotensin-Converting-Enzyme (ACE) Inhibitor—Trandolapril

ACE inhibitors reduce oxidative stress, improve endothelial dysfunction, and help to delay the progression of retinopathy and nephropathy. Trandolapril was compared to placebo in 41 normotensive patients with T1DM or T2DM and mild neuropathy over 12 months [45]. Peroneal MNCV increased in the Trandolapril group (38.3 ± 4.1 to 39.4 ± 4.1, *p* = 0.03) compared to a decrease in the placebo group (37.4 ± 4.8 to 36.6 ± 5.4, *p* = 0.03), and the sural nerve amplitude increased in the Trandolapril group (8.3 ± 6.4 to 10.0 ± 7.8, *p* = 0.04) and decreased in the placebo group (6.1 ± 6.3 to 5.6 ± 5.8). The peroneal M-wave amplitude (*p* = 0.03) and F-wave latency (*p* = 0.03) increased in the Trandolopril group with no change in vibration-perception threshold, cardiac autonomic function, neuropathy symptom or deficit score [45].

### 3.8. Fatty Acids: γ-Linolenic Acid (GLA)

GLA is a vital component of the microcirculation and neurons, and those with diabetes have a reduced ability to convert dietary linoleic acid to GLA, which may result in DPN [46]. In a clinical trial comparing GLA to placebo over 1 year [47], the placebo group demonstrated a significant decline in peroneal motor NCV (−1.86 ± 0.99 m/s), median sensory NCV (−2.14 ± 1.02 m/s) and sural sensory nerve amplitude (−0.96 ± 0.65 µV).

### 3.9. C-Peptide

C-peptide deficiency may contribute to the development of DPN [21]. Patients with type 1 diabetes and DPN (*n* = 250) were randomised to weekly subcutaneous placebo (*n* = 106) or C-peptide 0.8 mg (*n* = 71) or 2.4 mg (*n* = 73), and the modified Toronto clinical neuropathy score (mTCNS), bilateral sural NCV and vibration perception threshold (VPT) at the great toe were assessed at 26 and 52 weeks [21]. VPT improved by 25% with C-peptide compared to placebo (*p* < 0.0001). Sural NCV increased in patients on C-peptide (+1.0 ± 0.24 m/s) but also increased in the placebo group (+1.2 ± 0.29 m/s). There was a small decline in the sural nerve amplitude (−0.2 ± 0.2 µV) and mTNCS (−1.02 ± 0.3) in the placebo groups [21].

**Table 1:** Summary of clinical trial outcomes of disease-modifying therapies and placebo in patients with DPN ^1^.

Study	Country	Drug	N (Total)		Multi-Centre (M) vs. Single Centre Study (S)	Outcome of the Study	Placebo Group Outcome
			Control	Drug			
Wahren et al. [21]	Multiple	C-Peptide	106	144	M	↑SNCV, ↑VPT, ↔ mTNCS ↔ SNAP	↑SNCV, ↑MNCV ↓SNAP
Ziegler et al. [22]	Multiple	A-lipoic acid	227	233	M	↔ NIS-LL + 7, ↔ peroneal MNCV, ↔ SNAP	↔ NIS-LL + 7, ↔ peroneal MNCV, ↔ SNAP
Brooks et al. [25]	Australia	Ruboxistaurin (PKCI) 32 mg	11	9	M	↔ SkBF ↔ nerve conduction parameters	↑peroneal NCV
Vinik et al. [27]	Multiple	Ruboxistaurin	68	137	M	↔/↑ VDT, ↔/↑ NTSS	?
Sorbinil Retinopathy Trial [29]	-	Sorbinil	103	89	-	↓DSP, ↔ median nerve sensation + motor measures, ↑ peroneal nerve NCV, ↔DN early clinical signs and symptoms	↓DSP, ↓peroneal MNCV
Jennings et al. [30]	United Kingdom	Sorbinil (250 mg)	6	8	-	↔/↑ AER, ↔ MCBMT, ↔ neurophysiology, ↓IVPR to collagen + ADP	↓AER, ↔ MCBMT, ↑ IVPR to collagen + ADP
O’Hare et al. [31]	United Kingdom	Sorbinil	10	21	-	↔ Metabolic control, ↔ neuropathy severity, ↔self-assessed symptom scores, 4 neuropathic ulcers developed, ↔ clinical manifestation, ↔ neurophysiology	↔ Metabolic control, ↔ neuropathy severity, 1 neuropathic ulcer developed
Bril et al. [32]	Canada	Ranirestat	134	415	M	↑ motor NCV	↔ NCV
Greene et al. [33]	United States of America	Zenerestat	50	158	M	↑ NCV, ↑nerve fibre density	↓ sural sensory MNFD, ↑ HbA1c, ↓ neurophysiology
Brown et al. [34]	United States of America	Zenerestat	472	956	M	↑/↔ NCS	↓NCS, ↓QST
Laudadio et al. [35]	United States of America	Ponalrestat	211	213	M	↔NCS	↓toe VPT, ↓Valsalva ratio,
Sundkvist et al. [36]	Sweden	Ponalrestat (600 mg)	99	216	M	↔VPT, ↔ NCV, ↔ NAPA, ↔ 30:15 ratio	↔VPT, ↔ NCV, ↓ 30:15 ratio
Ziegler et al. [37]	Germany	Ponalrestat	21	39	-	↔HRV, ↔ E/I ratio, ↔ symptoms, ↔ neurophysiology	?
Hotta et al. [38]	Japan	Fidarestat 1 mg	102	90	M	Mostly ↑ neurophysiology, ↑ symptoms	↔/↓ neurophysiology
Santiago et al. [39]	United States of America	Tolrestat (200 mg/400 mg)	192	180	M	↑MNCV, ↑ toe sensation, ameliorated pain	↓ MNCV
De Grandis et al. [40]	Italy	Acetyl-L-Carnitine (Levacecarnine)	166	167	M	↑ NCV, ↑ NCA, ↑ VAS	↑ VAS
Apfel et al. [41]	United States of America	rhNGF	515	504	M	↑ GSA, ↑ in 2 PBQ domains, ↔ NIS	↔ NIS
Bravenboer et al. [42]	Netherlands	ORG2766	32	30	-	↑VPT	?
Relanovic et al. [44]	Croatia	Thioctic acid (α-lipoic acid	20	90	M	↑ NCS	-
Malik et al. [45]	United Kingdom	Trandolapril	23	23	S	↑ Peroneal MCV, ↑M-wave amplitude, ↓F-wave latency, ↑sural nerve action potential amplitude	↔ VPT, ↔ Autonomic function, ↔ NSDS
Keen et al. [47]	United Kingdom	ɣ-Linolenic acid	57	54	M	↑MNCV, ↑ SNAP, ↑CMAP, ↑hot and cold thresholds, ↑ sensation, ↑ tendon reflexes, ↑ muscle strength	-

^1^ ADP, adenosine diphosphate; AER, albumin excretion rate; Country, country study conducted in; CMAP, compound muscle action potential; ↓, declined; DN, diabetic neuropathy; Drug, name of drug under investigation; DSP, distal symmetric polyneuropathy; E/I, The longest R-R interval during expiration and the shortest R-R interval during inspiration; GSA, global symptom assessment; HRDB, heart rate deep breathing; HRV, heart rate variation; IVPR, in vitro platelet responsiveness; 30:15 ratio, measure of heart rate reaction to standing; MCBMT, muscle capillary basement membrane thickness; MNCV, motor nerve conduction velocity (s); MNFD, myelinated nerve fibre density; NAPA, nerve action potential amplitude; NCA, nerve conduction amplitude; NCS, nerve conduction studies; NCV, nerve conduction velocity; NIS, neuropathy impairment score; NIS-LL + 7, neuropathy impairment score—lower limbs and seven neurophysiologic tests; NSDS, neuropathy symptom and deficit scores; NTSS-6, Neuropathy Total Symptom Score-6;PBQ, Patient Benefit Questionnaire; PKCI, protein kinase C inhibitor; QST, quantitative sensory testing; rhNGF, recombinant human nerve growth factor; SkBF, skin microvascular blood flow; SNAP, sensory nerve action potential, SNCV, sensory motor nerve conduction velocity (s);VAS, VAS pain score; VDT, vibration detection threshold; VPT, vibratory perception threshold; ↑, improved; ↔, no change; ↓, declined; -, not stated; ?, unclear.

**Table 2:** Demographic characteristics of studies ^1^.

Author	Year	Trial Length (Weeks)	Overall Participant Total	Male	Female	Ethnicity	Age in Years Mean (SD)	Aetiology of Diabetes	HbA1c (%)
O’Hare et al. [31]	1988	60	31	NS	NS	NS	NS	NS	NS
Jennings et al. [30]	1990	104	14	NS	NS	NS	NS	NS	NS
Ziegler et al. [37]	1991	52	60	33	27	NS	PL: 46.9 + 2.5, TX: 52.8 + 1.3	IDDM + NIDDM	PL baseline: 9.1 ± 0.3, Range 7.3–12.2, PL at 4 weeks 8.5 ± 0.3, PL at weeks 13–529.5 ± 0.4 TX baseline: 9.5 ± 0.2, Range 7.0–12.7%, Tx at 4 weeks: 9.2 ± 0.3, TX at weeks 13–52: 9.2 ± 0.2.
Sundkvist et al. [36]	1992	78	315	246	69	NS	PL: 48 ± 11, TX: 45 ± 12, Total 46 ± 12	Insulin treated + non-insulin treated	Baseline TX 8.79 ± 2.25. Baseline PL: 8.79 ± 2.17
Santiago et al. [39]	1993	52	372	289	83	NS	PL: 57.9 ± 10.5, Range 25–78; TX: 58.1 ± 10.9, Range 26–76	IDDM + NIDDM	PL mean 6.8 ± 1.2, range 4.1–11.2; TX: 6.7 ± 1.1, range 3.5–9.4
Sorbinil Retinopathy Trial [29]	1993	208	192	NS	NS	NS	18–56	IDDM	NS
Keen et al. [47]	1993	52	111	81	30	NS	PL: 52.9 ± 11.4, TX: 53.3 ± 11.1	NS	PL 9.6 ± 2.2, TX 9.7 ± 2.2
Bravenboer et al. [42]	1994	52	62	39	23	NS	PL: 47.1 + 10.7, TX: 47.5 + 12.8	IDDM	PL 9.7 ± 2.3, TX 9.0 ± 2.5
Malik et al. [45]	1998	52	46	46	0	NS	PL: 48·2 ± 11·0 TX: 48·7 ± 11·6	T1DM or T2DM	TX 10.1 ± 2.02; PL 10.8 ± 1.16
Laudadio et al. [35]	1998	78	424	NS	NS	NS	18–65 IC	Conventional insulin/oral hypoglycaemic agents/dietary control	IC 6.8–15.0
Relanovic et al. [44]	1999	102	110	28	37	NS	PL: 57.3 ± 6.4, TX (600 mg): 58.1 ± 17.3, TX (1200 mg): 58.0 4 ± 5.5	T1DM + T2DM	Baseline: PL 93 4 ± 2.2, TX (600 mg) 88 ± 1.5, TX (1200 mg) 9.1 4 ± 2.2 At 102 weeks: PL 9.14 ± 2.4, TX (600 mg) 9.2 ± 2.2, TX (1200 mg) 8.0 ± 1.5.
Greene et al. [33]	1999	52	208	127	81	NS	PL: 52.0 ± 1.7, TX (300 mg): 53.4 ± 1.4, TX (600 mg): 50.0 ± 1.7, TX (1200 mg): 52.8 ± 1.8	T1DM or T2DM	PL: 10.3 ± 0.3, TX (300 mg): 10.0 ± 0.3, TX (600 mg): 11.2 ± 0.2, TX (1200 mg) 10.4 ± 0.3
Apfel et al. [41]	2000	52	1019	643	376	NS	Baseline—PL 55.8 ± 10.4, Range 19–74, TX: 55.1 ± 11.3, Range 22–75	T1DM (26%) or T2DM (74%)	PL mean 8.7 ± 1.8), TX 8.8 ± 1.8
Hotta et al. [38]	2001	52	192	109	83	NS	PL: 56.7 ± 0.7; TX: 57.3 ± 0.9	Type 1—PL: 5 (4.9%), TX: 4 (4.4%). Type 2—PL: 97 (95.1%), TX: 86 (95.6%)	Baseline PL 7.9 ± 0.2; at 52 weeks 7.9 ± 0.2. Baseline TX 7.7 ± 0.1; at 52 weeks 7.9 ± 0.1
De Grandis et al. [40]	2002	52	333	NS	NS	NS	NS	NS	NS
Brown et al. [34]	2004	52	1428	872	556	White 1185, Hispanic 92, Black 91	PL: 51.9 ± 10.3, TX low dose: 52.9 ± 9.8, TX high dose: 52.5 ± 9.7	T1DM or T2DM (N = 1161)	PL: 7.7 ± SD1.5 (range 4.8–11.7), TX low dose 7.8 ± 1.7 (4–12), TX high dose 7.8 ± 1.5 (4–12.4)
Vinik et al. [27]	2005	52	205	122	83	NS	Total: 45.6 ± 8.41	T1DM + T2DM	Total: 8.8 ± 1.49
Brooks et al. [25]	2008	52	20	4	14	NS	PL 47.8 ± 10.7; TX 51.6 ± 7.6	T1DM or T2DM	PL 7.0 ± 1.2; TX 7.4 ± 1.5
Bril et al. [32]	2009	52	549	342	207	NS	Total: 55.6 ± 9.0	T1DM or T2DM	Total: 8.3 ± 1.4
Ziegler et al. [22]	2011	208	460	302	152	NS	Baseline—PL 53.9 ± 7.6, TX: 53.3 ± 8.3,	344 participants with T2DM, 110 participants with T1D	Baseline PL.8 ± 1.9, Baseline TX 8.9 ± 1.8
Wahren et al. [21]	2016	52	250	137	113	NS	PL 47.1 ± 1.2, TX 46.1 ± 1.1	T1DM	PL: 7.9 ± 0.1, TX: 7.8 ± 0.1

^1^ HbA1c, glycated haemoglobin; IC, IC used where actual data not available; IDDM, insulin-dependent diabetes patients; NIDDM, non-insulin dependent diabetes patients; ns, not significant; NS, not stated; PL, placebo group; TX, treatment group.

## 4. Discussion

This systematic review highlights a potential explanation for the universal failure of clinical trials assessing disease-modifying therapies in DPN to identify meaningful outcomes [48]. Our analysis identifies a major flaw in the application of NCS-based assessment as a tool and the use of NCV as a primary endpoint in clinical trials. We find no evidence to support the assumption that NCV would decrease by 0.5 m/s/year in the placebo group, which raises concern about the validity of the statistical power calculations used in the previously reported clinical trials. Indeed, NCV shows considerable variability in its magnitude and direction of change when assessing placebo-associated change. Confounding factors influencing this variability could not be addressed due to limited demographic data, missing datapoints and inconsistency in the way that endpoints were reported in these studies. Furthermore, the clinical heterogeneity of the patients incorporating differing ethnicities and mixed aetiologies of diabetes (T1D and T2D) may have further confounded outcomes. Components of the metabolic syndrome (hypertension, dyslipidaemia, obesity, etc.), inflammation and different stringencies of glycaemic control will all affect nerve conduction parameters and contribute to DPN in those with T2D [49,50].

All 21 studies utilised NCS as a primary or co-primary study endpoint for DPN. However, small fibres constitute 70–90% of peripheral nerve fibres, conveying pain and thermal sensation and regulating sweat, tissue blood flow, inflammation, and wound healing [51]. Indeed, studies have demonstrated that significant small-fibre abnormalities may exist despite normal NCV in subjects with diabetes [51]. Shabeeb et al. concluded that the best method for quantitative evaluation and diagnosis of DPN was electrophysiology-based [52]. However, the implementation of a valid diagnostic test such as nerve electrophysiology does not necessarily translate to a robust (primary) endpoint in clinical trials in assessing the impact of an intervention.

A variety of other assessment tools may be considered. For example, sural nerve biopsy studies have previously demonstrated that unmyelinated nerve fibre damage precedes myelinated nerve fibre damage in DPN [53]. However, nerve biopsy is an invasive procedure requiring a specialised laboratory and expertise for quantification, which restricts its use in clinical trials of DPN [54]. In contrast, skin biopsy is a minimally invasive procedure which allows quantification of intra-epidermal nerve fibres [55,56] and the intra- and inter-observer variability for the evaluation of intra-epidermal nerve fibre density shows good agreement [56,57], and an international investigator consortium collated a normative database of intra-epidermal nerve fibre density [58]. Skin biopsy is advocated alongside an assessment of typical symptoms and sensory evaluation for the diagnosis of small-fibre neuropathy [30] and is also recommended as an endpoint in clinical trials [32]. Unfortunately, the availability of facilities to undertake skin biopsy and assess intra-epidermal nerve fibres is limited. Another technique that shows promise is corneal confocal microscopy (CCM)—a non-invasive, reproducible test which detects small nerve fibre loss in diabetic neuropathy [59,60], reliably [61] and with high sensitivity and specificity [62,63] and is comparable to intra-epidermal nerve fibre density [63,64]. CCM also predicts incident DPN [65] and detects nerve regeneration in people with DPN [66]. It has all the attributes of an ideal endpoint to identify early neuropathy, define at-risk individuals and monitor the progression or improvement of diabetic neuropathy [67]. An increasing number of interventional studies have shown that CCM can identify early nerve fibre regeneration following simultaneous pancreas and kidney transplantation [68], bariatric surgery [69], and GLP-1 [70] or insulin [71], as well as Cibinetide [72,73] and Omega-3 [74,75], which importantly precedes any improvement in symptoms, signs, and NCS. Importantly, endpoints such as CCM have demonstrated early nerve repair, whilst symptoms and NCV have not altered in interventional trials of pancreatic/kidney transplantation and Omega-3, and should thus be considered as a viable and reliable alternative [71,74,75]. Further studies are warranted to evaluate the efficacy of CCM to predict clinically meaningful improvements in people with DPN undergoing disease modifying interventions.

We acknowledge publication bias and English language bias as a limitation of this study. The clinical trials were small, generally poorly conducted, and had significant heterogeneity in their reporting and nerve conduction study protocols, a major confounding factor.

## 5. Conclusions

Nerve conduction studies have shown marked variability in the direction and magnitude of change of NCV in the placebo arm of multiple clinical trials of disease-modifying therapies for DPN; these findings are at odds with the assumed constant rate of annual decline suggested. These findings challenge the continued application of NCS-related measures as a primary endpoint in clinical trials of disease-modifying therapies for DPN, as endorsed by the FDA. This measure overlooks a growing body of evidence that suggests that small nerve fibre regeneration should be assessed in clinical trials of DPN, which may identify early nerve repair and demonstrate therapeutic efficacy of pathogenetic therapies for DPN, which, with other techniques, may erroneously be discounted. With this is mind, measures using corneal confocal microscopy, a rapid non-invasive and reiterative technique which can quantify small nerve fibre repair (thus fulfilling all FDA criteria), could act as a primary endpoint in clinical trials of DPN [76].

## Data Availability

Referenced literature can be found within the MEDLINE database.
